# Incident heart failure and the subsequent risk of progression to end stage kidney disease in individuals with type 2 diabetes

**DOI:** 10.1186/s12933-024-02279-y

**Published:** 2024-06-15

**Authors:** Sylvia Liu, Jian-Jun Liu, Keven Ang, Janus Lee, Clara Chan, Resham L. Gurung, Huili Zheng, Justin Tang, Su Chi Lim

**Affiliations:** 1 Clinical Research Unit, Khoo Teck Puat Hospital, Singapore, 768828 Singapore; 2 Cardiovascular and Metabolic Disorders Signature Research Program, DUKE-NUS Medical School, Singapore, 169857 Singapore; 3 Department of Medicine, Khoo Teck Puat Hospital, Singapore, 768828 Singapore; 4 Saw Swee Hock School of Public Heath, National University of Singapore, Singapore, 117549 Singapore; 5 Lee Kong Chian School of Medicine, Nanyang Technological University, Singapore, 308232 Singapore; 6 Diabetes Centre, Khoo Teck Puat Hospital, Singapore, 768828 Singapore

**Keywords:** Heart failure, End stage kidney disease, Type 2 diabetes

## Abstract

**Background:**

Diabetic kidney disease is an established risk factor for heart failure. However, the impact of incident heart failure on the subsequent risk of renal failure has not been systematically assessed in diabetic population. We sought to study the risk of progression to end stage kidney disease (ESKD) after incident heart failure in Asian patients with type 2 diabetes.

**Methods:**

In this prospective cohort study, 1985 outpatients with type 2 diabetes from a regional hospital and a primary care facility in Singapore were followed for a median of 8.6 (interquartile range 6.2–9.6) years. ESKD was defined as a composite of progression to sustained eGFR below 15 ml/min/1.73m2, maintenance dialysis or renal death, whichever occurred first.

**Results:**

180 incident heart failure events and 181 incident ESKD events were identified during follow-up. Of 181 ESKD events, 38 (21%) occurred after incident heart failure. Compared to those did not progress to ESKD after incident heart failure (*n* = 142), participants who progressed to ESKD after heart failure occurrence were younger, had higher HbA1c and higher urine albumin-to-creatinine ratio at baseline. The excess risk of ESKD manifested immediately after heart failure occurrence, persisted for two years and was moderated thereafter. Cox regression suggested that, compared to counterparts with no heart failure event, participants with heart failure occurrence had 9.6 (95% CI 5.0- 18.3) fold increased risk for incident ESKD after adjustment for baseline cardio-renal risk factors including eGFR and albuminuria. It appeared that heart failure with preserved ejection fraction had a higher risk for ESKD as compared to those with reduced ejection fraction (adjusted HR 13.7 [6.3–29.5] versus 6.5 [2.3–18.6]).

**Conclusion:**

Incident heart failure impinges a high risk for progression to ESKD in individuals with type 2 diabetes. Our data highlight the need for intensive surveillance of kidney function after incident heart failure, especially within the first two years after heart failure diagnosis.

**Supplementary Information:**

The online version contains supplementary material available at 10.1186/s12933-024-02279-y.

## Introduction

Diabetic kidney disease (DKD) is the leading cause of end stage kidney disease (ESKD) in many countries [1]. Intensive treatments on clinical risk factors and administration of kidney protective agents such as renin-angiotensin system (RAS) blockers, sodium- glucose cotransporter-2 (SGLT2) inhibitors and nonsteroidal mineralocorticoid receptor antagonists may favourably alter the trajectory of DKD progression. However, even within the controlled environment of clinical trials, residual risk of ESKD persists [2, 3]. This residual risk is particularly pronounced due to the complex interplay between diabetes and cardiovascular health. Notably, patients with diabetes face a substantially elevated risk of heart failure, with rates two to four folds higher compared to non-diabetic counterparts [4, 5],.

The inter-dependent interaction between kidney dysfunction and heart failure has been increasingly recognized [6]. A large body of evidence support that kidney dysfunction, manifested as a low glomerular filtration rate (GFR) and/or albuminuria, is a strong predictor of incident heart failure in both diabetic and non-diabetic populations [7–10]. However, only a very small number of studies have assessed the association between incident heart failure and the subsequent risk of progression to ESKD [11]. These studies were conducted in general population [12, 13], or in patients with advanced kidney disease under specialist care [14]. To our knowledge, only one prospective study has addressed the relationship between incident heart failure and risk of ESKD in diabetic population [15]. However, that study was a post hoc analysis of a clinical trial on erythropoiesis-stimulating protein in patients with type 2 diabetes, anaemia and chronic kidney disease (eGFR between 20 and 60 ml/min/1.73m^2^) [15]. In this context, we sought to study the risk of progression to ESKD after incident heart failure in individuals with type 2 diabetes and a broad spectrum of baseline kidney function.

## Methods

### Participants and follow-up

The ongoing SMART2D (Singapore Study of Macro-angiopathy and Micro-vascular Reactivity in Type 2 Diabetes) cohort study focuses on macro and microvascular complications in South East Asian patients with type 2 diabetes. We recruited 2057 outpatients from a regional hospital and an adjacent primary care facility in northern Singapore between 2011 and 2014 [16]. Type 2 diabetes was diagnosed by attending physicians after excluding type 1 diabetes and diabetes attributable to specific causes. Patients with uncontrolled hyperglycaemia (point-of-care fasting plasma glucose < 4.5 or > 15.0 mmol/L or HbA1c > 12%), and those with cancer, infectious disease and autoimmune disease on active treatments were excluded. We also excluded patients with CKD attributable to primary glomerulonephritis, systemic autoimmune diseases and specific genetic causes such as polycystic kidney disease from cohort enrolment. Participants were passively followed by reviewing their electronic medical records in a centralized data repository every two years. The repository contains routine outpatient records, hospitalization discharge summary, biochemical and imaging examinations, surgical and other interventional procedures. Participants were also invited for in-person research visit in the hospital every three years. Data from routine clinical care and research visits were combined to ascertain cardio-renal events. Additionally, we ascertained participant vital status by data linkage with national death registry. Follow-up was censored at 31 December 2021. We defined loss of follow-up as no respond to research visit invitation or did not visit the hospital or its affiliated medical facilities for routine clinical care for > 1 year. The date of the last clinical visit was considered the date of loss to follow-up.

### Exposure and clinical outcome

The primary exposure was incident heart failure which was diagnosed by attending physicians and ascertained by reviewing medical records according to the following criteria, (1) N-terminal prohormone B-type natriuretic peptide (NT-proBNP) > 125 pg/mL and, (2) evidence of heart failure from transthoracic echocardiography, with documentation of clinical symptoms and/or signs (European Society of Cardiology criteria) [17, 18]. Heart failure was subtyped into preserved ejection fraction (HFpEF) and reduced ejection fraction (HFrEF) based on left ventricular ejection fraction (LVEF ≥ 50% and < 50%, respectively). The clinical outcome was progression to ESKD which was a composite of, (1) progression to eGFR < 15 ml/min per 1.73m^2^ with at least one confirmation measurement 3 months apart or, (2) initiation of dialysis and sustained at least 3 months or, (3) death attributable to renal causes, whichever occurred first. Renal death was identified according to the primary cause of death on death certificate. According to our early study [19], we did not identify participants receiving kidney transplants during the follow-up up to December 2021.

### Clinical and biochemical variables

Ethnicity, sex, smoking status and duration of diabetes were self-reported. History of atherosclerotic cardiovascular disease (ASCVD) which included myocardial infarction and stroke was self-reported and validated by reviewing medical records after cohort enrolment. Blood pressure was measured three times using a semi- automated blood pressure monitor and the average was used. Mean arterial pressure (MAP) was calculated as (2x diastolic pressure + systolic pressure)/3. HbA1c was quantified using a point-of-care analyser (DCA Vantage Analyzer, Siemens, Germany). High density lipoprotein (HDL) cholesterol, low density lipoprotein (LDL) cholesterol and serum triacylglycerol were measured using enzymatic methods (Cobas C system; Roche Diagnostics, GmbH, Mannheim, Germany). Creatinine was measured by an enzymatic method that was traceable to isotope dilution mass spectrometry reference. The estimated glomerular filtration rate (eGFR) was calculated based on serum creatinine using 2019 Chronic Kidney Disease Epidemiology Collaboration (CKD-EPI) formula. Urine albumin was quantified using an immunoturbidimetric assay (Roche Cobas c, Roche Diagnostics, Mannheim, Germany). Albuminuria was presented as albumin-to-creatinine ratio (ACR).

### Statistical analysis

Clinical variables were presented as mean ± standard deviation (SD), median (interquartile range, IQR), or proportion where appropriate. Urine ACR and plasma triacylglycerol were natural log-transformed due to skewed distribution. Between-group differences in baseline clinical and biochemical variables were compared using chi-square and Student *t* tests.

We handled incident heart failure as a time-varying exposure (Additional File 1: Figure S1). Specifically, in those with heart failure occurrence during follow-up, the time from heart failure diagnosis onwards contributed to ‘exposure’ category whilst the time from cohort enrolment to heart failure diagnosis contributed to ‘non- exposure’ category [13]. For participants with events of ESKD but with prior incident heart failure, time from cohort enrolment to ESKD was taken as ‘exposure’. In those with neither ESKD nor incident heart failure, time from cohort enrolment to non-renal death or loss of follow-up or censor date was taken as ‘non-exposure’. We used Kaplan-Meier method to visualize the cumulative risk of ESKD after incident heart failure. Between-group difference was compared by log-rank test. We employed cause-specific Cox regression models to assess the risk of progression to ESKD after incident heart failure. The outcome was time from the date of heart failure diagnosis or cohort enrolment in the absence of heart failure to the date of ESKD, death, loss of follow-up or end of follow-up, whichever occurred first. The date of the first eGFR reading below 15 ml/min/1.73m^2^, date of dialysis initiation or date of renal death in ascertained ESKD events were considered as the date of ESKD event in the current analysis. Based on biological plausibility, we adjusted age, sex and ethnicity (Chinese as reference), diabetes duration, active smoking (yes or no), ASCVD history (yes or no), body mass index (BMI), MAP, HbA1c, HDL cholesterol, LDL cholesterol, triacylglycerol, RAS blocker usage (yes or no), insulin usage (yes or no), eGFR and urine ACR at baseline, i.e. cohort enrolment in multivariable model. Using the same approach as described above, we studied the risk of progression to ESKD after occurrence of HFpEF and HFrEF, respectively. We assessed the proportional hazard (PH) assumption by modelling heart failure X time as a multiplicative interaction term in the multivariable model and by Schoenfeld residual. Due to violation of PH assumption, we modelled incident heart failure as an exposure with time-varying coefficient.

As sensitivity analysis, we excluded participants with heart failure that occurred within 90 days before the ESKD because the diagnosis of heart failure might have been triggered by the symptomatic volume overload with rapid decline of kidney function [13]. We combined incident ESKD with non-renal death into a composite outcome to assess whether non-renal death might have affected the association between incident heart failure and the subsequent risk of ESKD as a competing risk.

Statistical analyses were performed using SPSS version 27 and R software version 4.0.5. A two-sided *p* value < 0.05 was considered statistically significant.

## Results

### Participant characteristics

After excluding participants with prevalent heart failure (*n* = 29) and ESKD (eGFR < 15 ml/min per 1.73m^2^, *n* = 43) at baseline, a total of 1985 outpatients with type 2 diabetes were included in this prospective study (Additional File 1: Figure S2). Participant baseline characteristics were presented in Table 1.

**Table 1:** Participant baseline clinical and biochemical characteristics

	All participants (*N* = 1985)	With incident heart failure (*N* = 180)	Without incident heart failure (*N* = 1805)	*P* value
Index age (years)	**57.3 ± 10.8**	**61.3 ± 9.3**	**56.9 ± 10.8**	**< 0.001**
Male sex (%)	50.7	50.6	50.7	0.961
*Ethnicity (%)*				**< 0.001**
Chinese	**51.2**	**35.6**	**52.8**	
Malay	**22.1**	**36.1**	**20.7**	
Asian Indian	**26.6**	**28.3**	**26.5**	
Diabetes duration (years)	**11.2 ± 9.0**	**13.6 ± 10.0**	**10.9 ± 8.8**	**0.001**
Active smoker (%)	8.6	9.6	8.5	0.611
ASCVD history (%)	**7.7**	**13.3**	**7.1**	**0.003**
Body mass index (kg/m^2^)	**27.7 ± 5.2**	**28.6 ± 5.4**	**27.6 ± 5.2**	**0.017**
HbA1c (%)	**7.8 ± 1.3**	**8.2 ± 1.6**	**7.7 ± 1.3**	**< 0.001**
HbA1c (mmol/mol)	**62 ± 10**	**66 ± 13**	**61 ± 10**	
*Blood pressure (mmHg)*				
Systolic pressure	**140 ± 19**	**147 ± 21**	**140 ± 18**	**< 0.001**
Diastolic pressure	79 ± 9	79 ± 9	79 ± 9	0.780
Mean arterial pressure	**100 ± 11**	**102 ± 11**	**99 ± 11**	**0.003**
*Lipid profile (mM)*				
HDL cholesterol	1.29 ± 0.35	1.25 ± 0.43	1.30 ± 0.35	0.053
LDL cholesterol	2.75 ± 0.82	2.78 ± 0.88	2.75 ± 0.82	0.709
Triacylglycerol (IQR)	**1.40 (1.04–1.94)**	**1.61 (1.12–2.13)**	**1.38 (1.03–1.92)**	**0.002**
*Baseline renal function*				
eGFR (ml/min/1.73m^2^)	**87 ± 25**	**77 ± 23**	**88 ± 25**	**< 0.001**
uACR (µg/mg, IQR)	**22 (7–93)**	**81 (18–458)**	**20 (6–79)**	**< 0.001**
*Medication usage (%)*				
Insulin	**28.2**	**47.2**	**26.3**	**< 0.001**
RAS blocker	**60.1**	**75.4**	**58.5**	**< 0.001**
Diuretics	**9.1**	**19.3**	**7.4**	**< 0.001**
Beta blocker	**9.1**	**17.6**	**7.4**	**< 0.001**

Data were presented as mean ± SD, median (interquartile range) or proportions. Between-group differences were compared by student *t* test, Mann-Whitney U test or X^2^ test where appropriate. ASCVD, atherosclerotic cardiovascular disease; eGFR, estimated glomerular filtration function; uACR, urine albumin-to-creatinine ratio; RAS, renin-angiotensin system. Variables differed significantly between groups have been highlighted in bold font

We identified 180 heart failure events during a median of 8.6 (IQR 6.2–9.6) years of follow-up (13,744 patient-years, crude incidence rate 1.31 per 100 patient-years). Compared to those with no events, participants with heart failure occurrence were older, had a longer duration of diabetes, higher BMI, HbA1c and systolic blood pressure. They also had a lower level of eGFR, higher urinary ACR and were more likely to be of Malay ethnicity and on insulin and RAS blocker treatments (Table 1).

### Risk of progression to ESKD after incident heart failure

We identified 181 ESKD events (174 progressed to sustained eGFR below 15 ml/min/1.73m^2^, 1 initiated maintenance dialysis before reaching CKD stage 5, and 6 death events were attributable to renal causes) during follow-up. Among them, 38 (21%) occurred after incident heart failure. Compared to those who did not progress to ESKD (*n* = 142) after heart failure, participants who progressed to ESKD after heart failure occurrence (*n* = 38) were younger, had higher HbA1c and higher urine ACR at baseline. They had no significant differences in baseline eGFR, blood pressure, CVD history, diabetes duration and demographic variables (Table 2).

**Table 2:** Baseline characteristics of participants with incident heart failure and subsequently progressed to ESKD versus those with incident heart failure but did not progress to ESKD

	Progressed to ESKD after incident heart failure (*N* = 38)	Did not progress to ESKD after incident heart failure (*N* = 142)	*P* value
Index age (years)	**57.2 ± 8.3**	**62.4 ± 9.3**	**0.002**
Male sex (%)	50.0	50.7	0.939
*Ethnicity (%)*			0.129
Chinese	26.3	38.0	
Malay	50.0	32.4	
Asian Indian	23.7	29.6	
Diabetes duration (years)	10 (7–20)	10 (5–20)	0.850
Active smoker (%)	8.1	10.0	0.728
ASCVD history (%)	10.5	14.1	0.567
Body mass index (kg/m^2^)	29.1 ± 6.7	28.5 ± 5.0	0.565
HbA1c (%)	**9.0 ± 1.6**	**8.0 ± 1.5**	**< 0.001**
*Blood pressure (mmHg)*			
Systolic pressure	151 ± 21	146 ± 20	0.200
Diastolic pressure	81 ± 8	78 ± 9	0.065
Mean arterial pressure	105 ± 11	101 ± 11	0.061
*Lipids profile (mM)*			
HDL cholesterol	1.17 ± 0.27	1.27 ± 0.46	0.204
LDL cholesterol	2.82 ± 0.89	2.77 ± 0.88	0.767
Triacylglycerol (IQR)	1.68 (1.15–2.82)	1.60 (1.11–2.04)	0.484
*Baseline renal function*			
eGFR (ml/min/1.73m^2^)	74 ± 28	78 ± 22	0.484
uACR (µg/mg, IQR)	**530 (100–1241)**	**44 (16–206)**	**< 0.001**
*Medications usage (%)*			
Insulin	59.5	44.0	0.093
RAS blocker	86.5	72.5	0.079
Diuretics	**32.1**	**16.5**	**0.020**
Beta blocker	21.4	21.0	0.944

Data were presented as mean ± SD, median (interquartile range) or proportions. Between-group differences were compared by student *t* test, Mann-Whitney U test or X^2^ test where appropriate. ASCVD, atherosclerotic cardiovascular disease; eGFR, estimated glomerular filtration function; uACR, urine albumin-to-creatinine ratio; RAS, renin-angiotensin system

**Fig. 1:**
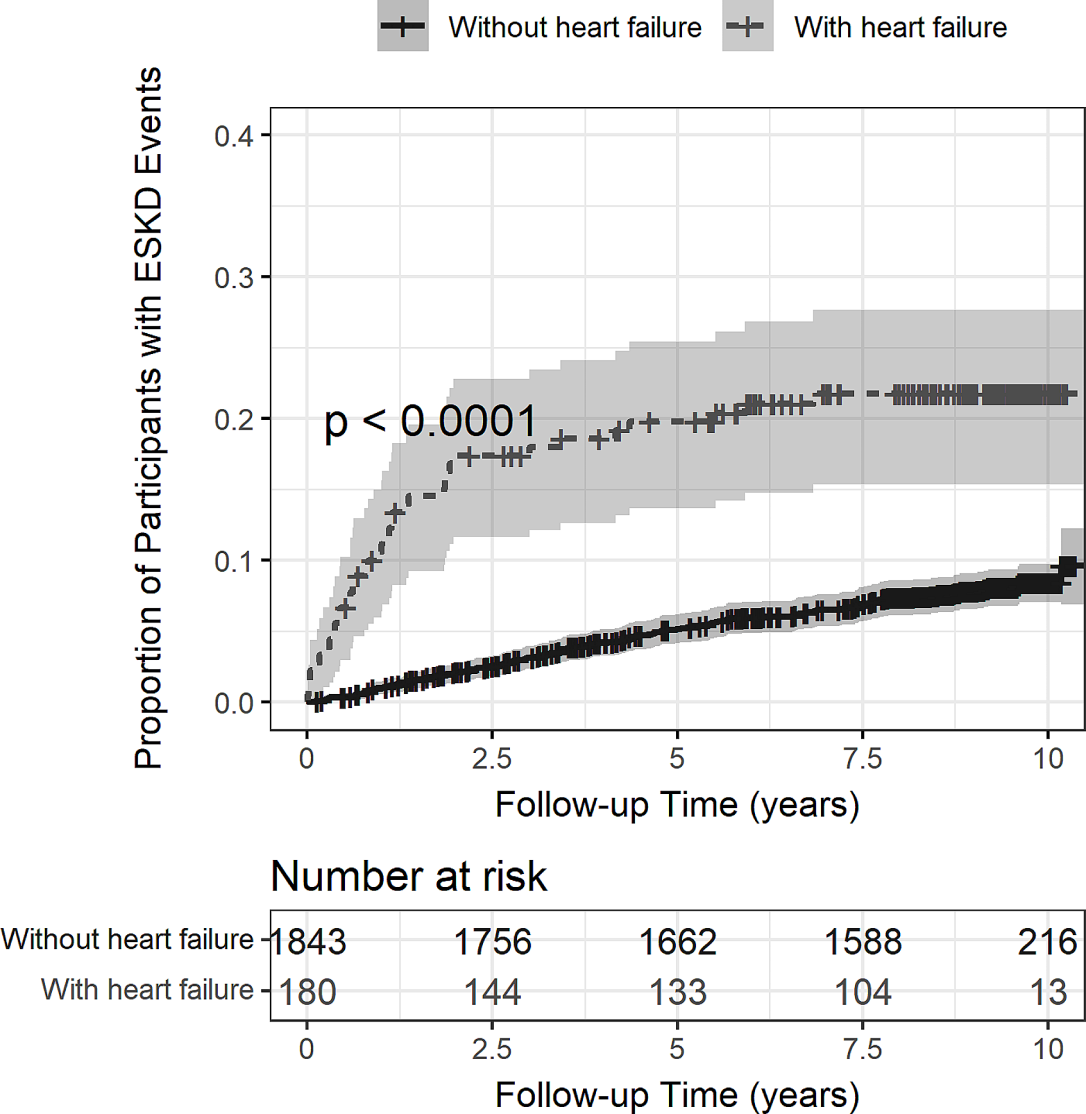
Risk of progression to end stage kidney disease in participants with and without incident heart failure during follow-up

As shown in Fig. 1, participants with incident heart failure had a significantly higher risk of progression to ESKD as compared to those without heart failure (log-rank *p* < 0.001). Compared to participants with no incident heart failure, the excess risk of ESKD in those with heart failure manifested immediately after heart failure occurrence, persisted for two years and was moderated thereafter as shown by the similar increment of cumulative ESKD events after two years. The median time from cohort enrolment to ESKD was 3.8 (IQR 1.9-6.0) years in those without heart failure events whereas the median time from heart failure occurrence to ESKD was 1.0 (IQR 0.4–1.9) year.

**Fig. 2:**
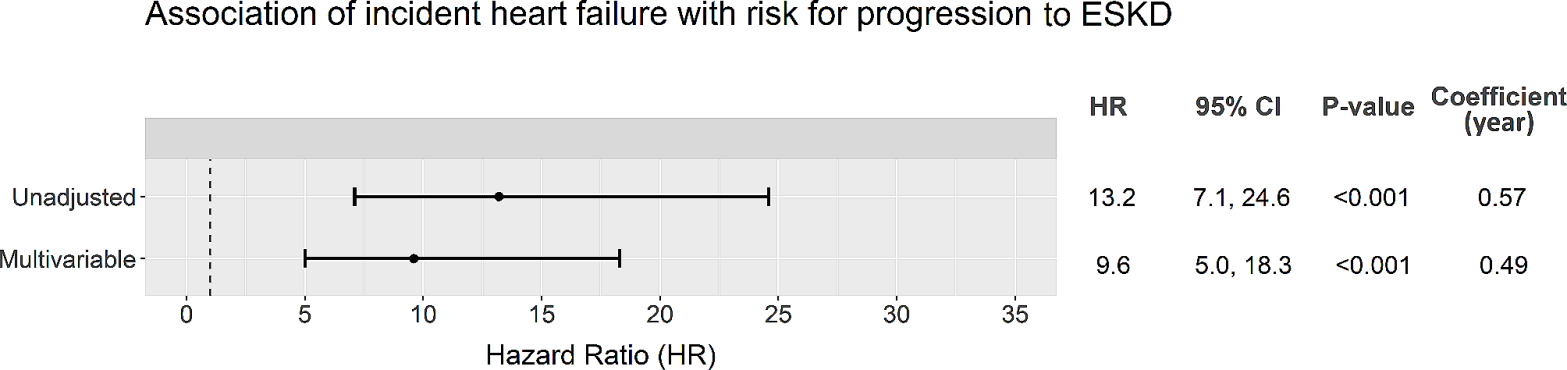
The association of incident heart failure with risk for progression to ESKD in univariable and multivariable Cox regression models. Multivariable model adjusted age, sex and ethnicity (Chinese as reference), diabetes duration, active smoking (yes or no), ASCVD history (yes or no), BMI, MAP, HbA1c, HDL cholesterol, LDL cholesterol, triacylglycerol (log-transformed), eGFR, urine ACR (log-transformed), RAS blocker usage (yes or no) and insulin usage (yes or no) at baseline (cohort enrolment). Incident heart failure was handled as a time-varying variable. It was modelled as a covariate with time-varying coefficient (per year) due to violation of proportional hazard assumption

Compared to those without event, participants with heart failure occurrence had an unadjusted 13.2 (95% CI 7.1–24.6) fold increased risk for progression to ESKD. The association remained statistically significant after adjustment for demographic and baseline cardio-renal risk factors including eGFR and urine ACR (adjusted HR 9.6, 95% CI 5.0–18.3, Fig. 2).

### Heart failure subtype and the subsequent risk of ESKD

**Fig. 3:**
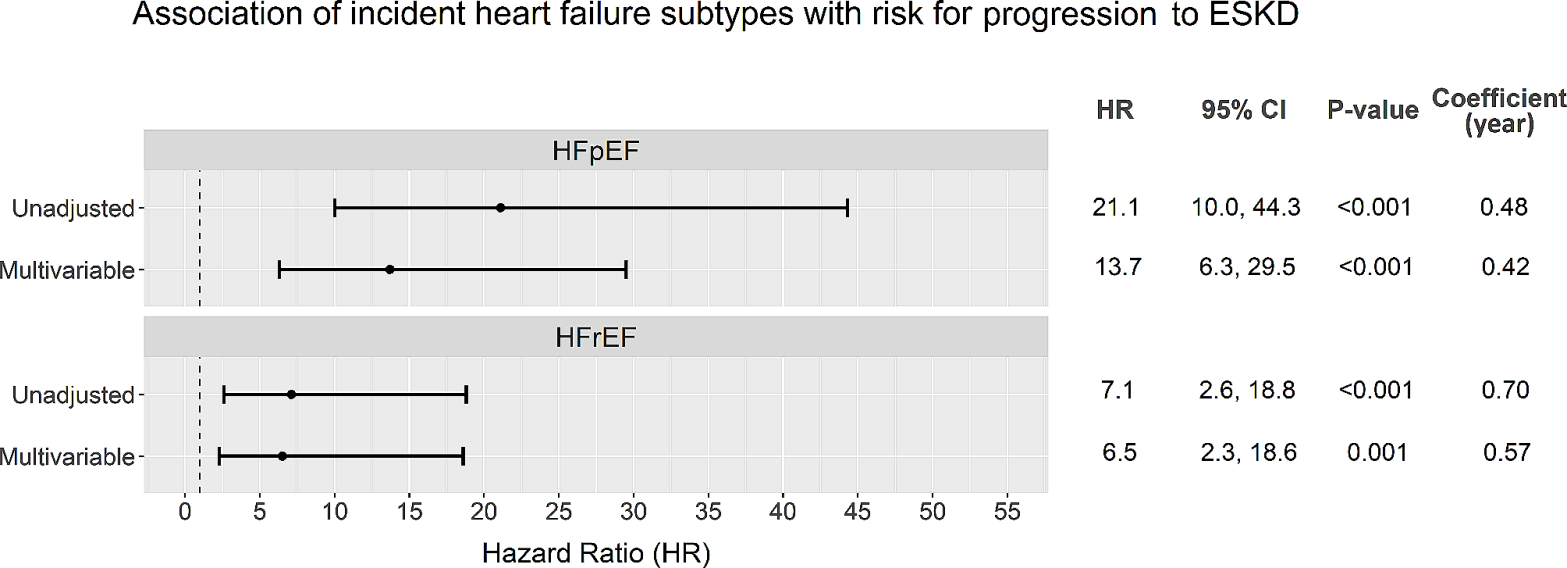
Association of heart failure subtype (incident HFpEF and HFrEF) with risk for progression to ESKD in univariable and multivariable Cox regression models. Multivariable model adjusted age, sex and ethnicity (Chinese as reference), diabetes duration, active smoking (yes or no), ASCVD history (yes or no), BMI, MAP, HbA1c, HDL cholesterol, LDL cholesterol, triacylglycerol (log-transformed), eGFR, urine ACR (log-transformed), RAS blocker usage (yes or no) and insulin usage (yes or no). Incident heart failure was taken as a time- varying variable. It was modelled as a covariate with time-varying coefficient (per year) due to violation of proportional hazard assumption

Of 180 heart failure events, 97 were classified as HFpEF, 68 were HFrEF while 15 had missing information for LVEF (Additional File: Table S1). The risk of progression to ESKD was significantly increased after both HFpEF and HFrEF. The excess risk of ESKD appeared more evident after HFpEF than that after HFrEF (adjusted HR 13.7 [6.3–25.9] versus 6.5 [2.3–18.6], Fig. 3).

### Sensitivity analyses

We excluded 7 participants with heart failure occurrence within 90 days before ESKD. The association between incident heart failure and the risk of ESKD remained statistically significant in both unadjusted and multivariable models (adjusted HR 7.2 [3.6–14.3], Additional File: Table S2).

We identified 118 death events not attributable to renal causes. Incident heart failure was significantly associated with the composite outcome of ESKD and non-renal death (adjusted HR 1.93 [1.44–2.58], Additional File: Table S3).

Additional adjustment for diuretics and beta blocker usage at baseline did not materially alter the association between incident heart failure and the subsequent risk of progression to ESKD in the multivariable model (Additional File: Table S4).

## Discussion

In this prospective study in individuals with type 2 diabetes, we found that, (1) more than 20% ESKD events occurred after incident heart failure, (2) compared to those with no heart failure events, the relative risk of progression to EKSD increased nearly ten folds after heart failure occurrence and, (3) the excess risk of ESKD manifested mainly in the first two years after heart failure. These data suggest that heart failure should be considered as an important precipitating factor for kidney disease progression in patients with type 2 diabetes.

Diabetes is the leading cause of ESKD whilst heart failure is the most common first presentation of cardiovascular disease in individuals with diabetes [20–22]. However, data regarding heart failure and the subsequent risk of ESKD in diabetic population are still scarce. To our knowledge, only one prospective study has addressed this question in the post hoc analysis of a clinical trial on erythropoietin in patients with type 2 diabetes, anaemia and chronic kidney disease [15]. The authors reported that 16.9% ESKD events occurred after incident heart failure, which was similar to our finding (21%) in diabetic patients with a broad spectrum of kidney function in real world setting. The authors also reported that the relative risk of ESKD was more apparent in a short period (30 days) after onset of heart failure, which was also generally agreeable with our observations in current study and a recent large study in non-diabetic population [12].

At least two plausible mechanisms may underpin the linkage between incident heart failure and the subsequent risk of progression to ESKD. First, the elevated central venous pressure resultant from heart failure may lead to renal venous hypertension, increased renal resistance, impaired intrarenal blood flow and, ultimately decline in filtration function [6, 23]. An early study showed that renal blood flow was dramatically decreased in patients with decompensated heart failure even in those with preserved filtration function [24]. Second, heart failure may activate sympathetic nervous system and increase the tone of renin-angiotensin-aldosterone system. These neurohumoral factors may worsen the extra- and intra-renal haemodynamic dysregulation and concomitantly increase inflammation, oxidative stress, endothelial dysfunction, and eventually drive the progressive loss of kidney function [25].

Consistent with some early studies in non-diabetic population [13], we observed a more apparent association of HFpEF with incident ESKD as compared to that of HFrEF (Fig. 3). We did not observe significant differences in history of ASCVD, blood pressure, eGFR and urine ACR between participants with incident HFpEF and HFrEF (Additional File: Table S1). Therefore, the stronger association between HFpEF and ESKD may not be explained by these traditional clinical risk factors. Myocardial injury associated with coronary artery disease or hypertension may be the predominant mechanism for HFrEF, whilst endothelial and microvascular dysfunction may play a major role in the pathophysiology of HFpEF [26–30]. It is reasonable to postulate that the differences in the severity of endothelial dysfunction or microvascular complication between diabetic patients with HFpEF and HFrEF may partly explain their differential susceptibility to CKD progression. On the other hand, insulin resistance and systemic inflammation are more strongly associated with HFpEF than HFrEF [31, 32]. Compelling evidence have shown that both insulin resistance and inflammation are drivers of kidney disease progression in diabetic population [33, 34]. Future clinical and preclinical studies are warranted to elucidate the pathophysiologic mechanisms underlying the stronger association between HFpEF and the subsequent risk of progression to ESKD in patients with type 2 diabetes.

Data from the current study have clinical implications. Together with evidence from the literature, our findings strongly support the notion that incident heart failure is an important precipitating factor for kidney disease progression in diabetic population. Clinicians should be aware of the high risk of progression to ESKD in patients with incident heart failure, intensively monitor their kidney function and minimize nephrotoxic exposure, especially in the first two years after onset of heart failure. Given that heart failure is often an unrecognized complication in diabetic population [4, 21], our work also highlights the importance of prevention, early detection and timely treatment of heart failure which may potentially reduce the risk of ESKD secondary to heart failure in patients with diabetes.

The strength of the study includes a relatively large sample size with a long- term follow-up. We took ESKD, i.e. the hard renal outcome instead of surrogates as study endpoint. We have considered the major cardio-renal risk factors in our data analysis and performed some sensitivity analyses to assess the robustness of our main finding. Nevertheless, a few important weaknesses should be highlighted. This is an observational study, we could not infer causality or elucidate pathophysiology. For example, the pathophysiologic mechanisms underpinning the more apparent linkage between HFpEF and risk of ESKD could not be addressed in this study. As that for all observational studies, unmeasured or residual risks are inevitable. We do not have information on comorbidities such as anaemia and socioeconomic status. Neither did we have data on medication usage during follow-up nor intensity and adherence to treatments after diagnosis of heart failure. Although no participants were treated by SGLT2 inhibitors at baseline, some of them should have been treated by this novel cardio-renal protective medication during follow-up. Similarly, we adjusted only cardio-renal risk factors measured at cohort enrolment in multivariable models for participants with and without events of incident heart failure because measurements of biochemical variables such as HbA1c, lipid profile and kidney function at the time of heart failure diagnosis were not available in our dataset. Therefore, analytical outcomes from multivariable models should be interpreted with caution. Additionally, the event numbers of HEpEF and HFrEF were relatively small in our study. Therefore, large- sized studies are warranted to further characterize the associations between heart failure subtype and risk of ESKD. Finally, Asian people are known to have high risk of ESKD as compared to European descents. Future studies are needed to assess whether findings from our Asian people with diabetes are generalizable to other ethnic groups.

## Conclusion

Incident heart failure impinged a high risk for progression to ESKD in individuals with type 2 diabetes. Intensive surveillance of kidney function is warranted after diagnosis of heart failure, especially in the first two years after heart failure occurrence.

### Electronic supplementary material

Below is the link to the electronic supplementary material.

Supplementary Material 1:  Description of data: Figure S1: analysing incident heart failure as time-varying exposure. Figure S2: participant selection; Table S1: baseline characteristics of participants with incident HFpEF and HFrEF; Table S2: Association of incident heart failure with risk for progression to ESKD after excluding heart failure events occurred within 90 days before ESKD; Table S3: Association of incident heart failure with the composite of ESKD and non-renal death; Table S4: Hazard ratios and 95% confidence intervals of all covariates after additional adjustment for usage of diuretics and beta blocker in the multivariable Cox regression model.

## Data Availability

The dataset generated/analysed during the study could not be shared publicly. However, anonymized data may be shared upon reasonable request from corresponding authors after obtaining approval from Singapore National Healthcare Group Ethic Committee.

## Abbreviations

ACR: Albumin-to-creatinine ratio
ASCVD: Atherosclerotic cardiovascular disease
BMI: Body mass index
CKD: Chronic kidney disease
CKD-EPI: Chronic kidney disease epidemiology collaboration
DKD: Diabetic kidney disease
eGFR: Estimated glomerular filtration rate
ESKD: End stage kidney disease
HFpEF: Heart failure with preserved ejection fraction
HFrEF: Heart failure with reduced ejection fraction
IQR: Interquartile range
LVEF: Left ventricular ejection fraction
MAP: Mean arterial pressure
NT-proBNP: N-terminal prohormone brain natriuretic peptide
RAS: Renin angiotensin system
SGLT2: Sodium- glucose cotransporter-2
