# Supplementary material for: Incident heart failure and the subsequent risk of progression to end stage kidney disease in individuals with type 2 diabetes
Source: Cardiovasc Diabetol. 2024 Jun 15;23:204. doi: 10.1186/s12933-024-02279-y (PMC11180396; doi:10.1186/s12933-024-02279-y)
Supplement: Supplementary file 1 — Supplementary Material 1: Description of data: Figure S1: analysing incident heart failure as time-varying exposure. Figure S2: participant selection; Table S1: baseline characteristics of participants with incident HFpEF and HFrEF; Table S2: Association of incident heart failure with risk for progression to ESKD after excluding heart failure events occurred within 90 days before ESKD; Table S3: Association of incident heart failure with the composite of ESKD and non-renal death; Table S4: Hazard ratios and 95% confidence intervals of all covariates after additional adjustment for usage of diuretics and beta blocker in the multivariable Cox regression model. [file 12933_2024_2279_MOESM1_ESM.docx]

**Incident heart failure and the subsequent risk of progression to end stage kidney disease in individuals with type 2 diabetes**

Sylvia Liu, Jian-Jun Liu, Keven Ang, Janus Lee, Clara Chan, Resham L Gurung, Huili Zheng, Justin Tang, Su Chi Lim

**Supplementary Figure and Tables**

**Figure S1: incident heart failure was analysed as a time-varying exposure**

**
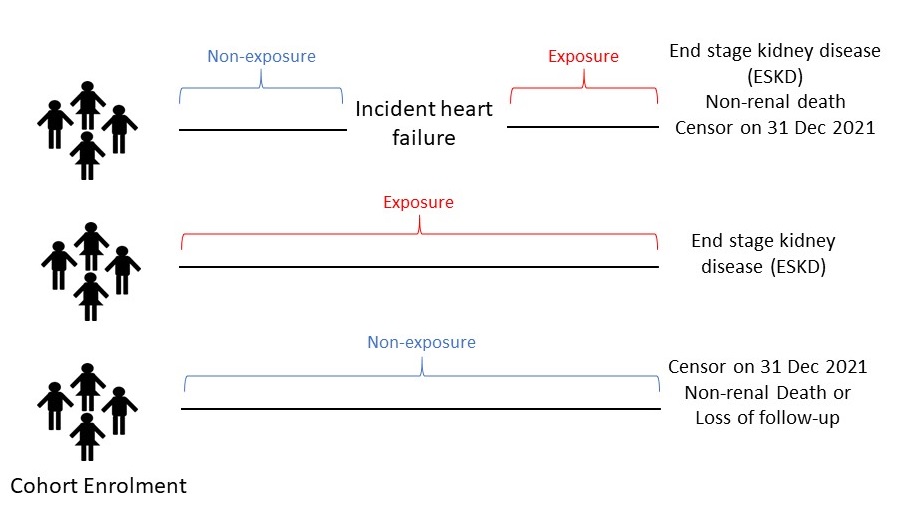
**

**Figure S2: Participant selection**

**
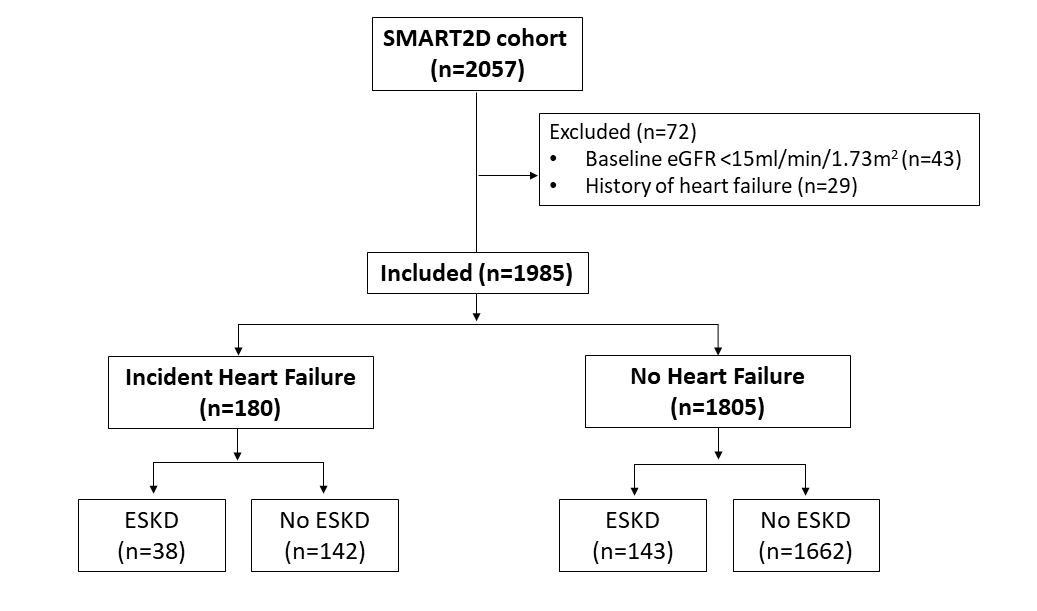
**

**Table S1: baseline characteristics of participants with incident HFpEF and HFrEF**

|  | HFpEF  (N=97) | HFrEF  (N=68) | *P* value |
| --- | --- | --- | --- |
| Index age (years) | 62.1 ± 9.5 | 60.6 ± 9.5 | 0.309 |
| Female sex (%) | 62.6 | 29.0 | <0.001 |
| Ethnicity (%)  Chinese  Malay  Asian Indian | 33.3  40.4  26.3 | 39.1  30.4  30.4 | 0.417 |
| Diabetes duration (years) | 10 (5-20) | 14 (6-22) | 0.109 |
| Active smoker (%) | 6.1 | 14.7 | 0.065 |
| ASCVD history (%) | 11.1 | 15.9 | 0.361 |
| Body mass index (kg/m^2^) | 29.2 ± 5.9 | 28.2 ± 4.7 | 0.253 |
| HbA1c (%) | 8.0 ± 1.5 | 8.4 ± 1.7 | 0.104 |
| Blood pressure (mmHg)  Systolic pressure  Diastolic pressure  Mean arterial pressure | 149 ± 20  79 ± 10  102 ± 11 | 146 ± 22  80 ± 9  102 ± 12 | 0.269  0.405  0.829 |
| Lipids profile (mM)  HDL cholesterol  LDL cholesterol  Triacylglycerol (IQR) | 1.31 ± 0.50  2.78 ± 0.88  1.72 (1.15-2.39) | 1.16 ± 0.29  2.75 ± 0.88  1.56 (1.10-1.91) | 0.024  0.809  0.232 |
| Baseline renal function  eGFR (ml/min/1.73m^2^)  uACR (µg/mg, IQR) | 75 ± 25  99 (18-376) | 78 ± 22  57 (24-600) | 0.438  0.626 |
| Medications usage (%)  Insulin  RAS blocker | 41.8  74.7 | 55.9  77.9 | 0.075  0.635 |

**Table S2: Association of incident heart failure with risk for progression to ESKD after excluding heart failure events occurred within 90 days before ESKD**

|  | **HR (95% CI)** | ***P* value** | ***Coefficient (year)*** |
| --- | --- | --- | --- |
| Unadjusted | 9.7 (5.0-18.9) | <0.001 | 0.62 |
| Multivariable | 7.2 (3.6-14.3) | <0.001 | 0.53 |

Cox regression - time to ESKD as outcome. Incident heart failure was modelled with time-varying coefficient.

Multivariable model adjusted for age, sex, and ethnicity (Chinese as reference), diabetes duration, smoking (active versus others), ASCVD history (yes versus no), BMI, mean arterial pressure, HbA1c, HDL, LDL cholesterol, log-transformed triacylglycerol, usage of RAS blocker (yes versus no), insulin (yes versus no), baseline eGFR and log-transformed urine ACR.

**Table S3: Association of incident heart failure with the composite of ESKD and non-renal death**

|  | **HR (95% CI)** | ***P* value** |
| --- | --- | --- |
| Unadjusted | 3.89 (2.96-5.12) | <0.001 |
| Multivariable | 1.93 (1.44-2.58) | <0.001 |

Cox proportional hazard regression - time to the composite of ESKD and non-renal death as outcome (no violation of PH assumption was identified).

Multivariable model adjusted for age, sex, and ethnicity (Chinese as reference), diabetes duration, smoking (active versus others), ASCVD history (yes or no), BMI, mean arterial pressure, HbA1c, HDL, LDL cholesterol, log-transformed triacylglycerol, usage of RAS blocker (yes versus no), insulin (yes versus no), baseline eGFR and log-transformed urine ACR.

**Table S4: Association of incident heart failure with the subsequent risk of end stage kidney disease (ESKD) after additional adjustment for usage of diuretics and beta blocker in multivariable Cox regression model**

|  | Hazard ratio | 95% CI | *P* value |
| --- | --- | --- | --- |
| Incident heart failure (yes versus no) | 9.84 | 5.15-18.78 | <0.001 |
| time coefficient (per year) | 0.49 | 0.37-0.63 | <0.001 |
| Index age (years) | 0.96 | 0.94-0.98 | <0.001 |
| Male sex (male versus female) | 1.11 | 0.77-1.58 | 0.59 |
| Ethnicity  Chinese  Malay  Asian Indian | Reference  1.89  0.73 | 1.19-2.39  0.44-1.21 | 0.004  0.22 |
| Diabetes duration (years) | 1.00 | 0.98-1.02 | 0.80 |
| Active smoker (yes versus no) | 0.80 | 0.46-1.38 | 0.42 |
| ASCVD history (yes versus no) | 0.83 | 0.46-1.49 | 0.53 |
| Body mass index (kg/m^2^) | 0.98 | 0.95-1.01 | 0.22 |
| HbA1c (%) | 1.04 | 0.93-1.18 | 0.49 |
| Mean arterial pressure (mmHg) | 1.00 | 0.98-1.02 | 0.81 |
| Lipids profile (mM)  HDL cholesterol  LDL cholesterol  Triacylglycerol (natural log) | 0.79  0.96  0.93 | 0.45-1.41  0.79-1.17  0.65-1.32 | 0.43  0.70  0.68 |
| Baseline renal function  eGFR (ml/min/1.73m^2^)  uACR (natural log) | 0.967  1.72 | 0.961-0.974  1.55-1.90 | <0.001  <0.001 |
| Medications usage (yes versus no)  Insulin  RAS blocker  Diuretics  Beta blocker | 0.77  0.92  1.87  1.18 | 0.53-1.12  0.60-1.42  1.31-2.67  0.83-1.68 | 0.17  0.71  0.001  0.35 |

Multivariable Cox regression: time to incident ESKD as outcome. All variables in the table above have been included as covariates in the multivariable model. Incident heart failure was handled as a time-varying variable. It was modelled as a covariate with time-varying coefficient (per year) due to violation of proportional hazard assumption.
